# BJS Open 2024 Reviewer List

**DOI:** 10.1093/bjsopen/zraf028

**Published:** 2025-03-11

BJS Open thanks the following for being referees in 2024. Please forgive any typographic errors or omissions—the lists are as accurate and complete as possible given the numbers involved. Your time and efforts are appreciated by the journal and authors. Peer review requires that as many of us as possible participate in that process. If you would like to participate more, please contact us any time.

Eirik Kjus Aahlin

Muriel Abbaci

Abdelaziz Abdelaal

Stefan Acosta

Kaisa Ahopelto

Alberto Aiolfi

Takashi Akiyoshi

Daniel Aliseda

Martin Almquist

Roland E. Andersson

Rocio Anula

Knut Magne Augestad

Laura Alaimo

Elisa Bannone

Ugo Boggi

Josep M. Badia

Anita Balakrishnan

José M. Balibrea

Ayan Banerjea

Savio George Barreto

Fabian Bartsch

Lee Bayliss

Giulio Belfiori

Francesco Belia

Marie Irene Bellini

Melissa Bennett

Kasia Bera

Felix Berlth

Frederik Berrevoet

Janet E. Berrington

Luca Bertolaccini

Aneel A. Bhangu

Gabriel Birgand

Panagiota Birmpili

Ian Bissett

Olof Bladin

Michael R. Boland

Akseli Bonsdorff

Andreas Brandl

Giuseppe Brisinda

Kristoffer Watten Brudvik

Eleanor Burden

Anna Burelli

Sally Benton

Ronan Cahill

Nicholas Campain

Ferdinando Cananzi

Daniel Carradice

Catarina Carvalho

Anna Casey

Peter Cashin

Marco Catarci

Alice Cattelani

Adam Chambers

Prem Chana

George J. Chang

Leseman Charlotte

Guo-Ming Chen

Jason Chuen

Stefano P.B. Cioffi

Amila Cizmic

Cillian Clancy

Jennifer Cleland

Rachael E. Clifford

Simone Conci

James Coulston

Stefano Crippa

Jonathan Cubitt

Paul Cullis

Nathan Curtis

Guowei Che

Marie Dahl

Dimitrios Damaskos

Matteo De Pastena

Ashe S. DeBiasio

Marcello Di Martino

Sophie Dream

Cem Dural

Gert J. de Borst

Carlo de Pasqual

Hanna de la Croix

Barbora East

Brett Ecker

Åsa Edsander Nord

Florian K. Enzmann

Richard Evans

Gareth Evans

Csonger Fabo

Pietro Familiari

Iain Feeley

Luis Fernandez

Simon Fleming

Caterina Foppa

Rachel Forsythe

Pietro Fransvea

Asmund Fretland

Maximos Frountzas

Molly Fuchs

Sebastien Gaujoux

Daniel Gero

Anthony R. Glover

Mahesh Goel

Michel Gonzalez

Claire Goumard

Anna Løf Granstrøm

Sally Griffin

Ugo Grossi

Travis E. Grotz

Rachel Guest

Lucia Guilabert

Theophile Guilbaud

Brenig L. Gwilym

Mario Giuffrida

Georg M. Haag

Dieter Hahnloser

Julie Hallet

Naoki Hiki

Marianne Hollyman

Karoline E. Horisberger

John Houghton

Tom Hubbard

Sheena Hubble

Merlijn Hutteman

Jinwook Hwang

Povilas Ignatavicius

Matthias Ilmer

Ilkka Ilonen

Ian James

Ahsan Javed

Carl Jorns

Rathachai Kaewlai

Sivesh K.K. Kamarajah

Monish Karunakaran

Vikram Kate

Pankaj Kaul

Joonas Kauppila

Michael E. Kelly

Oleksandr Khoma

Cheesun Kim

Jong Won Kim

Bilal Kirmani

Ulla Klaiber

Dyre Kleive

Rory Kokelaar

Arto Kokkola

Laura Koskenvuo

Joanne Kotsopoulos

Tiuri E. Kroese

Knut J. Labori

Patrick Larsson

Kristoffer Lassen

Tessa Le Large

Hans Lederhuber

Matthew J. Lee

Yung Lee

Pål Dag Line

David S. Liu

Hao Liu

Dileep N. Lobo

Per Loftås

Leyre L. Poch

Zipeng Lu

Panagis Lykoudis

Laura Maggino

Tom Mala

Ranjit Manchanda

Christopher Mantyh

Georgios A. Margonis

Ashley Marumoto

Lisa Massey

Diane Mege

Emmanuel Melloul

Panu Mentula

Andrea Merrill

Jeremy Meyer

Peter Minneci

Eric Mirallié

Marc Miserez

Shahin Mohseni

Philip C. Müller

Vasi Naganathan

Masaya Nakauchi

Sandip Nandhra

David Naumann

Angelica Nepi

Nikolaj Nerup

Zi Ng

Olga Nilsson

Atsushi Oba

Masayuki Ohtsuka

Maria Olausson

Tomohiro Osaki

Mel Osborne

Rachel Palmer

Elena Panettieri

John Paoli

Do Joong Park

Stefano Partelli

Francesco Pata

Shraddha Patkar

Gianluca Pellino

Jose M. Pimiento

Mauro Podda

Irinel Popescu

Matthew A. Popplewell

Shelley Potter

Arfon G.M.T. Powell

Shaun Prentice

Pascal Probst

Philip H. Pucher

Andrea Ruzzenente

Mohamed Rabie

George Ramsay

Elena Rangelova

Jari Rasanen

Francesca Ratti

Keith Roberts

Pierluigi A. Romano

Didier Roulin

Martin Rutegård

Harm J. Rutten

Sami Alexander Safi

Mushegh Sahakyan

Athanasios Saratzis

Adele E. Sayers

Lorenzo Scardina

Marc D. Schwartz

David Scott-Coombes

Carolyn Seib

Olivia Sgarbura

Mostafa Shalaby

Joseph Shalhoub

Iestyn M. Shapey

Omair Shariq

N. Sharma

Anita Sharma

Eiji Shinto

Giuseppe Sica

Caroline Siew

Suvi Sippola

Ajith K Siriwardena

Henry George Smith

Jimmy Bok-Yan So

Ernesto Sparrelid

Antonino Spinelli

Thomas Stoop

Christian Sturesson

Zhonghua Sun

Robert Sutcliffe

Arul Edward Suthananthan

Katsuhito Suwa

Tsuyoshi Takahashi

Eider Talavera Urquijo

Karl Tamussino

Patricia Tejedor

Teun Teunis

Dinesh Thekkinkattil

Alexis Theodorou

Michael Torkzad

Hannah Travers

Frederic Triponez

James L. Turvill

Maureen Twiddy

Aki Uutela

Rahul Velineni

Diana Vetter

Alessandro Vitale

Charles M. Vollmer

Thibault Voron

Elvira Vos

Jony van Hilst

San Ming Wang

Imtiaz Wani

Ulrich Wellner

Tomas Wester

Esther Westwood

Jimme Wiggers

Richard Wood

Zhouqiao Wu

Sheraz Yaqub

Mohamed Yassin

Marina Yiasemidou

Alistair Young

Urs Zingg

Tobias Zingg

Jan Zmuc

Andres Zorrilla-Vaca

